# Better Fiscal Rules for a More Integrated EMU

**DOI:** 10.1007/s10272-022-1091-0

**Published:** 2022-12-16

**Authors:** Pierre Jaillet, Christian Pfister

**Affiliations:** 1 des relations internationales et stratégiques (IRIS), Jacques Delors Institute and Institut, 18, rue de Londres, 75009 Paris, France; 2 Science Po, 27 Rue Saint-Guillaume, 75337 Paris Cedex 07, France

**Keywords:** H60, H62, H63

## Abstract

The COVID-19 pandemic and the pressure it has created on member states’ public finances have led European authorities to suspend the EU’s fiscal rules. In fact, these rules had long been criticised by academics and had only been abided by exceptionally. Beyond the rules themselves, the lessons one can draw from the past 20 years also give good reason to revise the EU overall coordination framework.

In the general case, fiscal rules are justified as a result of a bias towards deficit in democracies (Wyplosz, 2012). Referring to rules also echoes the debate on rules vs discretion, with the adoption of rules decreasing uncertainty by fostering the transparency of policies and accountability of policymakers. In the specific case of monetary unions, an additional concern is to avoid negative externalities. Such externalities can arise via monetary policy, which can more easily surrender to fiscal dominance, or via financial market expectations, which can lead to contagion. It is worth noting that the notion of bias towards deficit, the advantage of rules for decreasing uncertainty and the risk of fiscal dominance are of little value for supporters of the Functional Finance Theory (FFT). Among them are authors like Blanchard (2021), Mathieu and Sterdyniak (2021) and followers of the so-called Modern Monetary Theory (MMT; see Drumetz and Pfister, 2021). In the FFT, fiscal dominance prevails spontaneously (discussed below) and financial markets have only a subsidiary role in determining interest rates, which makes it useless to reduce uncertainty.

* The views expressed are those of the authors and do not commit the Jacques Delors Institute, the IRIS, Paris 1 Panthéon Sorbonne or Sciences Po.

**Pierre Jaillet**, Jacques Delors Institute; and French Institute for International and Strategic Affairs (IRIS), Paris, France.

**Christian Pfister,** Sciences Po, Paris, France.

## The initial fiscal framework: Debated rules

Looking back at the context prior to the Maastricht Treaty, a few key points emerged from the discussions:

- the absence of a political union, excluding transfers between member states
- the independence of monetary policy from national budgetary policies, excluding the so-called monetary financing of public deficits, even though the latter was eventually allowed
- the assumption that national economies would spontaneously merge into a homogeneous monetary area
- a presumption of good conduct on the part of governments, allowing for a soft macroeconomic coordination based on peer pressure.

The fiscal framework could thus be confined to a prudential role consisting of ensuring the sustainability of public debts, avoiding the main externalities between partners and encouraging member states to preserve sufficient counter-cyclical room for manoeuvre to deal with cyclical developments or shocks.

This system quickly showed its limits, despite changes brought about between 2005 and 2015 (Regling, 2022), by adding a medium-term objective for the structural deficit (1% or 0.5% of GDP depending on the level of public debt) and requiring an annual reduction of one-twentieth of the part of public debt exceeding 60% of GDP. However, most member states did not comply with the rules (less than one in three times for France or Italy). Structural balances remained in most countries above the medium-term objective, with fiscal policies generally remaining pro-cyclical, and public debts did not follow the one-twentieth annual reduction rule, despite the continuous decline in interest expenditure.

Two important factors were at play. On the one hand, the macroeconomic surveillance carried out by the Commission, under tight intergovernmental control and mainly focused on public finance indicators, has not been able to prevent the macroeconomic drift of certain member states, particularly as regards their external deficits. On the other hand, market discipline has not played the stabilising role initially anticipated as a complement to fiscal rules.

However, if the fiscal framework has not fulfilled its original objectives, it is also because the assumptions on which the economic component of the Economic and Monetary Union (EMU) was based have not been validated: the dynamic of economic convergence, reflecting an optical illusion before the great financial crisis, reversed afterwards, with the eurozone becoming an increasingly heterogeneous area. Meanwhile, soft coordination and peer pressure have failed to prevent and correct deviant behaviours or to deliver a common economic policy orientation.

The relevance of the EU fiscal rules has also been questioned on three grounds:

*Limits on the possibility of stabilising the economy*. This limitation would follow from the underestimation of output gaps by the EU Commission (Darvas et al., 2018). However, Cette et Jaillet (2018) object that these estimation biases have not been shown as leading to the revisions in the structural fiscal balance. In fact, the revisions have remained limited between 0.5% and 1% of GDP for the core countries of the euro area between 2012 and 2017.

*The realism of the reference value for debt and of the adjustment speed to reach it*. Returning to the implementation of the rule defined in 2011 (a reduction of the spread vis-à-vis the reference value at a pace of one-twentieth per year) would imply excessive adjustments in some countries (Giavazzi et al., 2021). As a result, it has been suggested that the increase in debt between 2020 and 2022, linked to exceptional factors with regard to past developments (the COVID-19 pandemic and the war in Ukraine), be ratified. However, it is unfortunately likely that, as a result of climate change and the destruction of natural ecosystems related to deforestation, extreme climate events and pandemics will become more and more common in the coming years. They can thus not be considered exceptional factors.

*The contingent, if not arbitrary, nature of norms*. This would particularly be the case when the inequality $r < g$, where r is the real natural interest rate, identified with the real rate of interest on government bonds, and g the real rate of growth of the economy, prevails (Blanchard, 2021). Indeed, this configuration increases the fiscal room for manoeuvre, since it becomes possible to combine primary deficits and the stability, if not the reduction, of the debt ratio. Consequently, if a primary balance ratio is to be retained as a norm, it should equal $(r - g)$ times the observed debt ratio, instead of a given fixed level (below 0.5% or 1% depending on whether the debt ratio is above 60%), as in the present arrangement. However, one can make the point that the room for manoeuvre allowed by weak interest rates could be used to repay the debt. Furthermore, Wyplosz (2019) indicates that the inequality $r < g$ is not the norm among OECD countries and that $r - g$ is very volatile in these countries.

## Some important contextual elements

### Pressures in favour of debt mutualisation

Proposals to mutualise debt have taken two forms: a partial mutualisation and a financial innovation. Conversely, or as a complement to these proposals, some suggest that the no-bailout clause be made more credible.

Debt mutualisation is a proposal that, under the Eurobonds label, has been put forward when the sovereign debt crisis exploded (see e.g. Delpla and von Weizsäcker, 2010; Bofinger et al., 2011; Giavazzi et al., 2021). It is suggested that part of the member states debt would benefit from a joint guarantee from the EU countries or be transferred to a European body that would issue bonds to finance itself. The issue is that, beyond the temporary hype that a new synthetic asset would create, it is hard to see what the best-rated sovereign issuers would stand to gain. Furthermore, even if less well-rated issuers could enjoy better conditions on the newly issued (or guaranteed) bonds, their other issues could find themselves "juniorised" and thus made costlier. Lastly, the risk of a domino effect would be strengthened (Tirole, 2012).

Financial innovation (the European Safe Bonds (ESBies) or Sovereign bond-backed securities (SBBSs)) has been suggested by Brunnermeier et al. (2017). In this proposal, financial institutions would buy euro area sovereign securities at market prices, according to a key close to the one of the ECB capital, and finance themselves by issuing multi-tranches securities, with the senior tranche (SBBSs) presenting the same degree of risk as the safest sovereign bonds in the euro area. SBBSs raise issues that are similar to those raised by Eurobonds.

Making the no-bailout clause more credible aims at reducing the element of moral hazard weighing on the public finances of any country participating in a monetary union, especially when debt is mutualised. Proposals in that direction take three main forms. In the first form, sanctions are strengthened by extending the conditionality foreseen by the European structural and investment funds to the whole EU budget (European Fiscal Board, 2018) and by the issuance of junior sovereign securities in case the net expenditure norm is breached (Bénassy-Quéré et al., 2018; Darvas et al., 2018). In the second form, an orderly procedure for sovereign default (Gros and Mayer, 2010; Bénassy-Quéré et al., 2018) and a European Monetary Fund (Gros and Mayer, 2010) would be created or the role the European Stability Mechanism (ESM) would be strengthened (Bénassy-Quéré et al., 2018). The third form of measures is the setting of concentration charges imposed on banks' sovereign exposures (Bénassy-Quéré et al., 2018). In the latter case, if the measure produced the expected diversification outcome, contagion risks could be increased.

In fact, if the principle of debt mutualisation has not been officially enacted, it has been implemented, whereas the no-bailout principle has been set aside. This has been observed during the sovereign debt crisis with the creation of the ESM and during the COVID-19 pandemic with the launch of the NextGenerationEU programme.

## A monetary policy under heavy constraints

Regarding conventional monetary policy, within a framework (Blanchard, 2021; Mathieu and Sterdyniak, 2022) where potential growth would have been curtailed by overly restrictive economic policies, neutral rates would have become lower than the rate of growth. They would also be lower than the lowest safe interest rate monetary policy can achieve, thus invalidating monetary policy as a stabilising device, hence the return of FFT. Indeed, according to FFT, the role of the budget is to stabilise the economy, pursuing both full employment and price stability. The role of monetary policy is then only to guarantee the limitless financing of fiscal deficits and to administer the interest rates on public securities at low levels (Drumetz and Pfister, 2021).

An implication regarding monetary policy in a monetary union without a fiscal union such as the euro area is that in comparison with the present institutional arrangements, FFT implies the re-nationalisation of the objective of price stability. Consequently, Mathieu and Sterdyniak (2022) suggest that in the framework of a coordination exercise of fiscal policies, member states should present strategies to help reach the euro area inflation objective. However, this would imply a weakening of the role played by the EU, since it is currently the ECB that is in charge of pursuing price stability at the euro area level, and, as shown below, could not be achieved without central bank support.

Unconventional monetary policies can take different forms, implying an increasing degree of integration with fiscal policy (with the second one debatable and the third one unacceptable):

*The monetary financing of public debt*. Contrary to a widely held opinion, monetary financing by the central bank is not prohibited by the Treaty on the Functioning of the European Union, which only prohibits the direct extension of loans to public administrations and the purchase of public securities on the primary market. This has allowed the ECB to conduct its public asset purchase programmes.

*The administration of longer-term interest rates*. This administration can itself take two forms. In the first form, it aims at reducing sovereign spreads, as in Mario Draghi's "whatever it takes" pledge in July 2012. In that direction, Blanchard (2021) suggests the central bank should intervene in case of pure sunspots in markets for public securities, without specifying the basis on which this diagnosis would be made or who would be responsible for it. In the second form, possibly combined with the first one, the administration of long-term interest rates aims at the levels of rates; this is yield curve control. Mathieu and Sterdyniak (2022) recommend that the ECB should intervene in the sovereign debt markets to ensure that real rates stay below the rate of growth. Accordingly, the inequality $r < g$ could be maintained permanently. However, the ECB's reputation would be severely impaired. In both forms, there would be a risk that the central bank could be trapped by financial market participants. Any event affecting in particular the most indebted countries would be dramatised, precipitating the intervention of the central bank and creating profitable arbitrage opportunities.

*A fiscal role for the central bank*. Examples include proposals for helicopter money (Martin et al., 2021); MMT, where the central bank systematically settles public expenditures under the instructions of the Treasury, giving it unlimited credit (Drumetz and Pfister, 2022); and the situation where a sovereign, especially if it is a large one, would default on its debt held by the central bank. In all these cases, the losses recorded by the central bank would trigger a loss of confidence in the currency and a very strong inflationary pressure, as repeatedly demonstrated by historical experience.

## A heterogeneous euro area

The reversal of the convergence dynamics after the great financial crisis seems to validate Krugman's (1993) thesis, according to which the economic and monetary integration area fosters the specialisation of economies and the polarisation of high value-added activities towards the areas initially best endowed with physical and human capital. Indeed, over the past decade, the southern European economies have lost the bulk of the ground gained before 2008, while the Central and Eastern European countries kept on catching up. Perhaps most worryingly, the major economies in the area have diverged after 2009, with income per capita of France and Italy falling in current euros by 13% and 17%, respectively, compared to Germany, according to World Bank data.

One can hardly blame the euro or budgetary rules for inefficient productive specialisations or trade underperformance, and national economic policies clearly incur a part of responsibilities. However, the growing heterogeneity of the euro area also appears to be largely attributable to the dysfunctional nature of the single market.

In this regard, intra-EU trade has stagnated since 2012 and a recent ECB study estimates the contribution of the changeover to the euro on intra-EU trade at only 5% between 1995 and 2015 (Gunella et al., 2021). Even worse, financial integration has remained the blind spot of EU policies and surveillance. Intra-area capital flows declined after the financial crisis and even more so after the euro area crisis of 2010-2012. Banking systems and financial markets remain fragmented, which fosters the spontaneous polarisation of activities, puts pressure on national fiscal policies and hinders the transmission channels of the single monetary policy (Jaillet and Vidon, 2018). Various studies have indeed highlighted the complementarity of private/public risk sharing in monetary unions (see, for example, Farhi and Werning, 2017) as well as the cushioning role of financial integration in the United States (Melitz and Zumer, 2002). That role is marginal in the euro area, with fiscal transfers remaining embryonic. The burden of absorbing shocks and the costs related to the lack of convergence are thus borne solely by the budgets of the member states and the ECB, which partly explains the failure of the European budgetary framework.

### Risks related to the war in Ukraine

The conflict between Russia and Ukraine weighs on the macroeconomic situation and the public finances of the member states. On the basis of the revisions to the ECB and the EU Commission economic forecasts between December 2021 and June 2022, the war would have led to a reduction of 1.5 percentage points in the GDP growth rate forecast for the euro area in 2022, with substantial downward risks. Furthermore, most governments have, to various degrees, adopted measures supporting households' income to shelter them from the peak of inflation resulting from the war and the shortages it has created in the food and energy sectors but also in other parts of the economy. The fiscal costs of these measures can for instance be evaluated at 2% of GDP in France. On top of these compensation measures, new and sizeable discretionary public expenditure will add up, with notable rises in military expenditure (for example, Germany plans to increase this by more than €100 billion over the next few years) and in investments aimed at strengthening the energy autonomy of Europe while respecting its climate change commitments. Beyond that, the participation in the reconstruction of Ukraine, a country whose vocation is to join the EU in the future, will also weigh on the member states' public finances. Overall, the war in Ukraine induces the governments in the euro area to postpone even more of their fiscal adjustments. As such, the EU fiscal rules have been suspended until 2024, while the Commission has revised its forecast for the euro area cyclically adjusted public deficit in 2022 by close to four percentage points since last year.

## Recent proposals for reforms

### The questioning of quantitative norms

In most proposals for amendments to the fiscal rules, the 3% reference value for the deficit is given up in the case of countries that do not fulfil the debt reference value. As this is the case for most euro countries, the following discussion focuses on the debt criterion. Indeed, the criticisms concerning the limitations allegedly put on the possibility of stabilising the economy and on the lack of realism of the debt reference value converge towards two sorts of proposals: reducing the pace for reaching the 60% reference value, thus making the latter a long- if not a very long-term objective, or suppressing quantitative rules altogether.

In the first sort of proposals, the principle of an objective for the debt ratio is kept and paired with a norm for the increase in net new public expenditure. For instance, the European Fiscal Board (2018) has made such a proposal, also keeping the objective of reducing the debt ratio to 60% of GDP. It has then amended this proposal during the COVID-19 pandemic, by differentiating the adjustment speed towards the target, so that the time horizon for reaching the reference value could exceed 25 years in the case of a very indebted country such as Italy (European Fiscal Board, 2020). Bénassy-Quéré et al. (2018), Darvas et al. (2018), Giavazzi et al. (2021) and Martin et al. (2021) all propose similar rules. In these proposals, the possibility to enact new tax measures that would raise the expenditure ceiling would in fact give member states a very wide degree of discretion, with the appending risk of depriving the surveillance procedure of any content. Finally, the ESM suggests maintaining the 3% reference value for the deficit, while raising the reference value for the debt to 100%, together with an expenditure rule allowing the reduction of the adjustment speed towards the targeted debt ratio. However, this proposal raises a credibility issue since raising the reference value would allow the anticipation of other future increases. With the view to offering greater flexibility to member states and increasing national ownership, the recent proposals made by the European Commission (2022) are very much along the same lines. Member states would be given more leeway in designing their own medium term fiscal plans, provided that they "respect a common EU framework". The Commission also advocates longer and more gradual adjustment paths.

Suppressing rules is suggested in two approaches. In the first one (Blanchard et al., 2021), rules are replaced with standards and a deficit would be considered as excessive if debt does not appear as sustainable with a high degree of probability, taking into account current and projected economic policies. In case the primary balance allowing the stabilisation of debt would not exceed a given threshold, for instance 5%, the sustainability standard would be considered fulfilled; in the opposite case, the country should adjust. In an even more radical approach, any quantitative norm or standard is eliminated in favour of a mere coordination of fiscal policies (Mathieu and Sterdyniak, 2021, 2022). For example, a country would be asked to change its fiscal stance only if it is demonstrated to be detrimental to its partners, e.g. because it would be considered overly restrictive.

### The issue of governance

Most of the aforementioned proposals entail important changes in the nature of the governance of the fiscal framework. An implicit assumption is that failure of the latter, beyond its design flaws, primarily lies with the European authorities and more specifically with the Commission. This reproach is largely unfounded, as member states constantly exert pressure on macroeconomic surveillance and blithely disregard their commitments. Intergovernmental logic prevails over community discipline.

Yet most of the proposals mentioned above advocate a kind of re-nationalisation of the fiscal framework, with targets on public expenditures defined under the responsibility of national governments and parliaments. The main risk this would create would be to further weaken the coherence of the euro fiscal framework by subjecting it to national political vagaries. Moreover, the issue of arbitrage would arise in the event of non-compliance with national commitments or conflicts of objectives between member states, while the Commission would be sidelined. Martin et al. (2021) propose entrusting independent national committees with the responsibility of validating the fiscal policy objectives and assessing their implementation. But it is far from clear how such advisory committees could achieve precedence over the political authorities. It would be up to the European Council of Ministers to validate the national commitments, but has it ever invalidated any? It would also be "heard" by the European Parliament. None of this seems particularly binding.

In the option of "budgetary standards" proposed by Blanchard et al. (2021), it would be up to committees of experts to set the trajectory of public debt. It would then be up to the Court of Justice of the European Union (CJEU) to rule in case of disagreement or conflict, which would inevitably lead to multiple disputes and lengthy procedures. Here too, the European economic authorities would be sidelined for the sake of a dialogue between experts and judges, with the risk of making the CJEU a scapegoat for national governments and public opinions.

### A specific treatment for some expenditures

Although this reflects the deliberate choice of member states, a reproach often addressed at fiscal rules is to have them postpone some growth-enhancing expenditures, such as investment expenditures, or some expenditures supporting "European public goods", such as the fight against climate change. It is thus proposed either to exclude these expenditures from the ceiling on net public expenditures, or to raise the debt ceiling by the same amount. The Commission takes up this proposal by suggesting a more gradual debt reduction path for countries that put forward a set of reform and investment commitments (European Commission, 2022).

However, many euro area countries are already very well endowed in public infrastructure, thus allowing for the reduction and redirection of public investment. Furthermore, the issue arises as to what can be considered an investment; if those expenditures are really productive, they should spontaneously benefit the countries that make them, both by increasing their GDP and by reducing their debt ratio. Finally, if the expenditures at stake contribute to European projects, there is no reason why they should not be financed at that level. Regarding the fight against climate change, it does not have to entail an increase in net public expenditure (Pfister and Valla, 2021).

## Conclusion

The minimalist "prudential" fiscal framework, which was supposed to guarantee the sustainability of public debts, limit externalities between economies and preserve governments' counter-cyclical margins of manoeuvre, has clearly become obsolete. However, fiscal rules remain justified in an EMU where the bulk of economic policies are the responsibility of national governments. Moreover, the new EU instruments – such as the ESM or the NextGenerationEU programme – are contingent public risk-sharing tools designed to cope with major shocks. They are not intended to play a role in cyclical stabilisation, and member states must therefore keep adequate capacities for action.

The new framework should abide by a few simple criteria: it should be based on common and non-manipulable, "opposable", numerical variables, and it should be transparent and easily controllable by the European authorities, which implies strengthening their prerogatives.

In light of these criteria, how could the new framework be articulated?

- The structural balance objective would become the central control variable. Its calibration (currently between 0.5% and 1% of GDP) could fall within a wider range – say between 0.5% and 1.5% – with national targets adjusted according to the level of debt.

- The estimation of structural balances would be the responsibility of the European Commission, supported by a committee of independent experts. The examination of the compliance of budgetary policies would take into account the in-year revision of output gaps.

- The 3% ceiling for the deficit would then no longer be justified, leaving adequate counter-cyclical space to member states.

- The evolution of structural balances should reflect the discretionary action of member states. It would be the net of debt interest charges but not of specific expenditure. Expenditure contributing to "European public goods" or with significant externalities, such as the fight against climate change, should be covered by programmes such as NextGenerationEU.

- The 60% GDP threshold for public debt is a long-term reference. Strict compliance with the structural balance objective, modulated according to the level of debt, would be the best guarantee of their sustainability. If this was deemed non-feasible, one option would be to raise the reference threshold to 100% while maintaining the current reduction rule (one-twentieth per year). Another option would be to maintain the 60% threshold while smoothing its trajectory (one-thirtieth per year).

However, the budgetary framework cannot be the scapegoat for all the dysfunctions of the EMU. It is not designed to ensure either the optimal coordination of economic policies or the coherence of its policy mix. Its overhaul must be part of a global reform of the EMU along two main lines: governance and convergence.

The strengthening of governance could take on two aspects. Firstly, as far as surveillance is concerned, the tools exist (SGP provisions supplemented by the European semester and the macroeconomic imbalance procedure), but their implementation is deficient. One remedy would be to merge the functions of the European Commissioner for Economy and of the President of the Eurogroup, with a real capacity for impetus and arbitration, as recommended in a report by the European Parliament (Böge and Bérès, 2017). In the same spirit, the Five Presidents' Report (Juncker et al., 2015) proposed the creation of a euro area treasury by 2025. Secondly, the European authorities would then be better equipped to ensure the coherence of the policy mix of the eurozone, which implies identifying a common fiscal policy (a fiscal stance in the face of the ECB's monetary stance) with which the member states would have to comply, also accepting symmetric macroeconomic adjustments. This naturally presupposes a political will to move towards a more federal model of governance. In that regard, the recent proposals made by the Commission mentioned above tend rather in the opposite direction, by strengthening national (and intergovernmental) prerogatives in managing the future fiscal framework (European Commission, 2022).

Without public risk sharing or adequate diversification of private risks, the cushioning of economic shocks and the costs of non-convergence are borne solely by member states' budgets and the ECB. The shortcomings of the single market thus explain to a large extent the difficulties of member states in complying with fiscal rules and the constraints faced by the single monetary policy. It is therefore illusory to introduce and enforce new common rules of the game in an increasingly heterogeneous economic area without strengthening governance. The current discussions on the fiscal framework should not overshadow the task of an overall EMU reform, beyond the reform of the fiscal framework.
